# Agriculture: Disaster-Ready Corn

**Published:** 2005-04

**Authors:** David A. Taylor

In the past 15 years, the number of droughts and other weather-related natural disasters worldwide has more than doubled, according to the international nonprofit Future Harvest. So it was welcome news when researchers at the University of California, Riverside, reported in the December 2004 *Plant Journal* what could be a way to improve the drought resistance of maize. Maize is now the most widely produced cereal in the world, having overtaken rice and wheat. Many areas where maize is grown, including parts of Africa, are vulnerable to drought.

Ethylene, a compound produced by plants, is believed to help plants adapt to stress, but can also cause leaves to wither in response to dry conditions. Daniel Gallie and colleagues found that the leaves of reduced-ethylene plants remained green longer than normal plants, and that reducing a plant’s ethylene production postponed withering and maintained leaf function.

Gallie and his colleagues identified transposons that had knocked out the enzyme that starts ethylene production in maize. Transposons are DNA elements that move from one position in the genome to another, knocking out the gene at the new position and replacing it. After screening thousands of plants, they found plants with mutant DNA affecting two of the three genes that make an enzyme needed for ethylene production. After confirming that the knockout mutants indeed produced less ethylene, they multiplied those plants and examined their growth in Riverside’s research fields. Not only did the mutant plants’ leaves stay green longer than normal plants, the plants’ leaves experienced higher-than-normal photosynthesis rates.

It’s still unknown whether the mutation improves the plants’ cereal productivity as well as leaf production, and how reduction of ethylene affects other plant functions. Jerry Cohen, deputy division director for Molecular and Cellular Biosciences at the National Science Foundation, says that in maize, ethylene also encourages rooting and adaptation to flooding; simply reducing ethylene in maize, he says, “could end up with nice plants lying on the ground.” Still, he adds, this study opens many new possibilities for maize improvement.

Mary Eubanks, a maize researcher at Duke University, notes that by providing better nutrition during drought, hardy maize would help maintain people’s immune systems and make them better able to resist infectious diseases. In the United States (the largest maize producer), hardier plants could reduce irrigation demand and consequent runoff. Irrigation represents more than half of the world’s freshwater demand, yet most of that is lost to evaporation, and runoff can pollute surface and groundwater with agricultural chemicals.

Gallie sees another potential benefit. With climate change, he says, competition between urban and agricultural demands on scarce water resources is likely to intensify. Any crop that uses less water can help ease that conflict. Cohen concurs: “Efficient use of water is the dominant theme of twenty-first century agriculture.”

